# A novel method for local clothing insulation prediction to support sustainable building and urban design

**DOI:** 10.1007/s00484-025-02934-3

**Published:** 2025-05-07

**Authors:** Junwei Lin, Ying Jiang, Yongxin Xie, Jianlei Niu

**Affiliations:** Department of Building Environment and Energy Engineering, The Hong Kong Polytechnic University, Hung Hom, Kowloon, Hong Kong

## Abstract

Clothing is crucial in thermal comfort evaluation, affecting heat exchange between the body and the environment. Assuming even clothing insulation across body segments can lead to inaccurate predictions, affecting building and urban design strategies. This study processed insulation data from 240 clothing ensembles to derive local insulation values. Regression models were developed to relate local and overall insulation values, allowing fast estimation of local insulation distribution for any given overall value. To validate the effectiveness of local clothing insulation values predicted by the proposed methods, measured real-time skin temperatures were collected from field experiments and compared with predicted values. Results demonstrated a significant accuracy improvement in the predicted local skin temperature from thermoregulation simulation combined with the proposed local clothing insulation estimation methods. The Jaccard Similarity Coefficient (JSC) increased by an average of 0.21, with body segments like the head, neck, shoulders, back, and arms showing nearly 0.4 or more improvement. These local insulation determination functions, used with a multi-nodal thermoregulation model, offer a simpler and more effective way to enhance thermal comfort assessment accuracy. By providing more precise local insulation values, these functions can help optimize building designs and urban planning strategies, leading to better thermal comfort for occupants.

## Introduction

The determination of clothing insulation plays a crucial role in evaluating thermal sensation and comfort (Fang et al. 2019; Singh et al. 2015). Accurate predictions of thermal sensation and comfort can contribute to improvement and design of both indoor (Zeng et al. 2024) and outdoor (Li et al. 2023) environment which is closely intertwined with health, well-being and energy efficiency (Lian 2024; Pérez-Fargallo et al. 2018). Physiological parameters such as skin temperature, which could benefit prediction accuracy (Zhang et al. 2010), are usually obtained from thermoregulation models which serve as an alternative to measurements. To meet the growing demand for higher-resolution thermoregulation modeling, advanced multi-nodal human body physiological models have been developed (Cheng et al. 2012), such as the Stolwijk model (Stolwijk 1971), the UTCI-Fiala multi-node model (Fiala et al. 2012), and the joint system thermoregulation model (JOS-3) model (Takahashi et al. 2021). Benefiting from these multi-nodal physiological models, thermal sensation and comfort models capable of providing predictions for non-uniform thermal environments, such as the UC Berkeley Comfort Model (Huizenga et al. 2001) and the UTCI model, have been developed. Unlike the traditional two-node models, these multi-nodal physiological models simulate the heat exchanges between different body segments separately. Accurate clothing insulation distribution is a critical component of multi-nodal models, which significantly impacts local skin heat exchanges with the surrounding environment. Ascertaining local clothing insulation distribution is accordingly imperative to ensure the reliability of thermoregulation simulation results across diverse environments.

One of the simplest methods to determine local clothing insulation values is to assume that the insulation values are evenly distributed across the whole body. This approach represents all local clothing insulation values with a single overall clothing insulation value. The overall clothing insulation can be easily accessed through tabulated values provided in thermal comfort-related standards (ASHRAE 2023; ISO 2009). Due to the availability of overall clothing insulation and inertia brought about by the widespread use of two-node models, which only allow an overall clothing insulation input, many researchers still choose the overall clothing insulation value as their simulation input, even with the latest multi-nodal models (Vanos et al. 2024; Völker and Alsaad 2018). In addition to habitual behavior, clothing data availability influences researchers'analysis choices. Clothing data from previous studies and datasets like the ASHRAE Global Thermal Comfort Database (De Dear 1998; Földváry Ličina et al. 2018) only include the overall clothing insulation when the multi-nodal models were not yet developed. As local clothing insulation datasets are limited and complex, research applied advanced multi-nodal models for simulation are forced to treat the entire body uniformly with a single overall clothing insulation value, even if this assumption may result in inaccurate predictions (Fojtlín et al. 2019). Some thermal comfort models, such as the physiological equivalent temperature (PET) (Höppe 1999), partially address this issue by distinguishing between covered and uncovered body parts. However, within covered regions, local clothing insulation values are still derived solely from overall clothing insulation, which can hardly capture variations between different body segments. As more multi-nodal models are proposed and adopted for practical applications, using an overall insulation value to represent the whole body becomes increasingly unreasonable and less efficient.

Researchers have developed several local clothing datasets in order to reduce inaccuracies caused by considering only the overall clothing insulation value. These established clothing datasets are based on the measurement results from thermal manikin (Lee et al. 2013; Nomoto et al. 2019; Tang et al. 2022), which is considered the accurate and reliable equipment to access local clothing insulation (Lei 2019). A few samples of local clothing insulation for typical clothing ensembles were provided in the test cases of multi-nodal thermoregulation models (Huizenga et al. 2001; Takahashi et al. 2021). With increased interest in local clothing insulation, several datasets testing a wide range of clothing ensembles have emerged. Lee et al. (2013) examined 40 typical clothing ensembles, measuring their local clothing insulation with a seated female-shaped thermal manikin ‘Monica’. Similarly, Tang et al. (2020) developed a clothing dataset with 35 typical winter clothing ensembles using an upright male-shaped manikin and later expanded it by testing 27 additional ensembles (Tang et al. 2022). ASHRAE also undertook significant projects to provide extensive detailed clothing data (Havenith et al. 2013; Smallcombe et al. 2022). In their 1504-TRP and 1760-TRP projects, the properties of 50 non-western and 69 western clothing ensembles were measured in both standing and sitting positions, supplementing the existing ASHRAE database (ASHRAE 2023). These recent studies enrich local clothing datasets and provide an opportunity to optimize the performance of advanced thermal comfort models.

Local clothing insulation values for the previously measured clothing ensembles can be readily obtained from existing clothing datasets, whilst estimation methods are needed for those clothing ensembles lacking data. To deal with unknown clothing ensembles, researchers have developed a few approaches to estimate local clothing insulation based on available measurement data. Nelson et al. (2005) proposed a method to estimate localized clothing insulation for the clothed body area using overall insulation data, though their predictions were not tailored for specific body segments. Havenith et al. (2012) introduced an adaptive clothing model, the UTCI-clothing model, insulation for the universal thermal climate index (UTCI). This model defines local clothing insulation as a linear function of ambient temperature. However, this model primarily accounts for environmental factors and does not consider individual variations in clothing choices. To improve local clothing insulation estimation, Tang et al. (2023) developed a set of linear regression models to predict segmented local clothing insulation values for individual garments and clothing ensembles. Their models utilize recorded effective clothing insulation values as the independent variable to predict the local clothing insulation values for garments from standards. The local clothing insulation distribution of an unmeasured clothing ensemble composed of these garments can subsequently be derived by summing the predicted insulation values of individual garments (Tang et al. 2022). For limited measured local clothing insulation values, these studies offer promising approaches to extrapolate the premeasured clothing data to unknown clothing ensembles using the regression method.

While the various approaches of local clothing insulation determination can benefit the implementations of advanced multi-nodal models, they have notable limitations. Relying on overall clothing insulation values, which are easily accessible, can lead to inaccuracies. To solve this issue, the published clothing datasets provide more detailed insulation values for different body segments but can hardly cover various clothing ensembles due to individual dressing styles. For unknown clothing ensembles, existing estimation methods, such as the UTCI-clothing model and Tang’s regression models, provide different approaches. The UTCI-clothing is primarily driven by environmental factors and may not fully capture individual clothing preferences. Tang's models transition the process of determining local clothing insulation from matching entire clothing ensembles to matching individual garments within existing datasets. Their models, however, still require users to document all garments involved and perform extensive data matching, processing and correction, which can be challenging to automate and time-consuming when handling large amounts of data. This process may also not be applicable for analyzing data collected from previous studies that only include overall clothing insulation values. To address these challenges, based on extensive clothing datasets, this study aims to establish regression models that directly predict local clothing insulation distribution based on easily accessible overall clothing insulation values, thereby simplifying and enhancing the estimation process. The specific objectives of this work include:

- To convert all insulation values in various clothing datasets to local clothing insulation and reorganize the processed data to be consistent and suitable for the advanced and widely used multi-nodal thermoregulation models.
- To develop a set of linear regression models that obtain local clothing insulation values of the clothing ensembles based on their overall clothing insulation values and compare these models to other estimation methods.
- To quantify the contribution of local clothing insulation to thermoregulation simulations and assess the validity of proposed estimation methods.

The local clothing insulation prediction methods proposed in this research are expected to contribute to the development and implementation of the thermoregulation model and support the follow-up evaluations of thermal stress and thermal comfort.

## Methodology

**Fig. 1:**
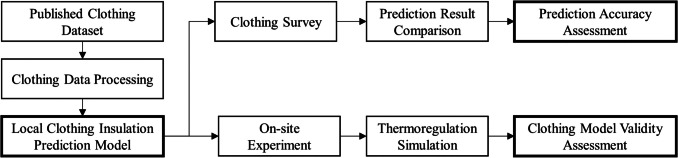
Schematic of the research workflow

In an attempt to develop and assess the local clothing insulation prediction methods, the procedures of this study include a clothing survey of human subjects, an on-site experiment for collecting subjects’ skin temperatures and thermal sensations, a regression analysis of clothing insulation data, and an assessment of the impact of estimated local clothing insulation values on thermoregulation simulations. The clothing survey data was used to validate the prediction results from the regression models while skin temperatures measured in the field experiment were utilized to assess the model accuracy when integrated into multi-nodal thermoregulation simulations. A schematic representation of the overall research workflow is provided in Fig. 1.

### Clothing survey

A clothing survey was conducted in this study to demonstrate how to implement the proposed local clothing insulation prediction methods and compare the prediction results with those from other estimation methods developed by Tang et al. (2023). In this survey, 205 participants (101 males and 104 females) from Beijing and Hong Kong, China were required to record their clothing choices over seasons. By combining data from these two regions with diverse climate conditions, the survey could capture a border range of clothing patterns. A short questionnaire was designed to collect the participants’ clothing choices. The questionnaire classified the garments into four categories including tops, bottoms, socks and shoes. Participants were asked to choose the types of garments most closely matching what they wore within each category. All the provided options of garments were based on the ASHRAE (ASHRAE 2023) and ISO (ISO 2009) standards, in which the corresponding effective clothing insulation (${I}_{clu}$) could be accessed. The sample of the questionnaire is shown in Appendix A.

Documenting the types of standardized garments can facilitate the comparison between different prediction methods. For methods proposed in this study, a key variable, the overall clothing insulation value for a participant, could be calculated by summing the effective clothing insulation values of all garments in the clothing ensemble, following Eq. 1 (ISO 2009):

1 $${I}_{cl}=\sum {I}_{clu,j}$$

where ${I}_{clu,j}$ refers to the effective clothing insulation of the garment $j$ and ${I}_{cl}$ refers to the overall clothing insulation of the clothing ensemble. The unit of clothing insulation is clo (1 clo = 0.155 m^2^·K/W). The proposed prediction methods then derived local clothing insulation for each body segment (${I}_{cl,i}$) from the overall clothing insulation:

2 $${I}_{cl,i}=f\left({I}_{cl}\right)$$

In Tang’s methods, the effective clothing insulation value of each garment (*j*) for each body segment (*i*) was first calculated, which is expressed as ${I}_{clu,i,j}$ (Eq. 3). As shown in Eq. (4), by summing the values of ${I}_{clu,i,j}$, the local clothing insulation value for each body segment could be obtained.

3 $${I}_{clu,i,j}=f\left({I}_{clu,j}\right)$$

4 $${I}_{cl,i}=f\left(\sum {I}_{clu,i,j}\right)$$

Based on these equations and the detailed clothing survey information, local insulation predictions across different methods could be easily accessed and used for comparison.

### Field experiment

The field experiment conducted in this study aimed to provide measured skin temperature for comparison with simulated data from thermoregulation models. The experiment was undertaken on the university campus in Hong Kong from June 2023 to August 2023. Considering the need of accurate thermal comfort assessment in the tropical regions such as in Hong Kong, the experiment focused on the summer period, prioritizing validation under hot conditions. A total of 34 subjects were recruited, and their basic information is listed in Table 1. Each subject participated in one to three times of experiments, resulting in a total of 86 human subject tests being completed. The field experiment has obtained research ethics approval by the PolyU Institutional Review Board (Reference Number: HSEARS20221023001).

**Table 1:** Characteristics of subjects recruited

	Male	Female	Total
Count	15	19	34
Age (years)	23.4 ± 2.7	23.2 ± 3.6	23.3 ± 3.2
Height (cm)	174.8 ± 6.9	166.5 ± 4.7	170.5 ± 7.1
Weight (kg)	71.5 ± 13.5	59.0 ± 9.8	64.7 ± 13.1
Body fat (%)	17.7 ± 6.4	27.1 ± 6.9	22.8 ± 8.1
Overall clothing insulation (clo)	0.3 ± 0.1	0.3 ± 0.1	0.3 ± 0.1
Metabolic rate (met)	1.0		
Posture	sitting		

**Fig. 2:**
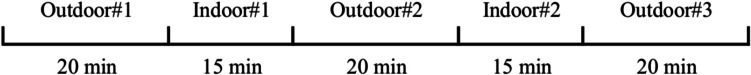
Experimental procedure that alternated indoor and outdoor environments

**Fig. 3:**
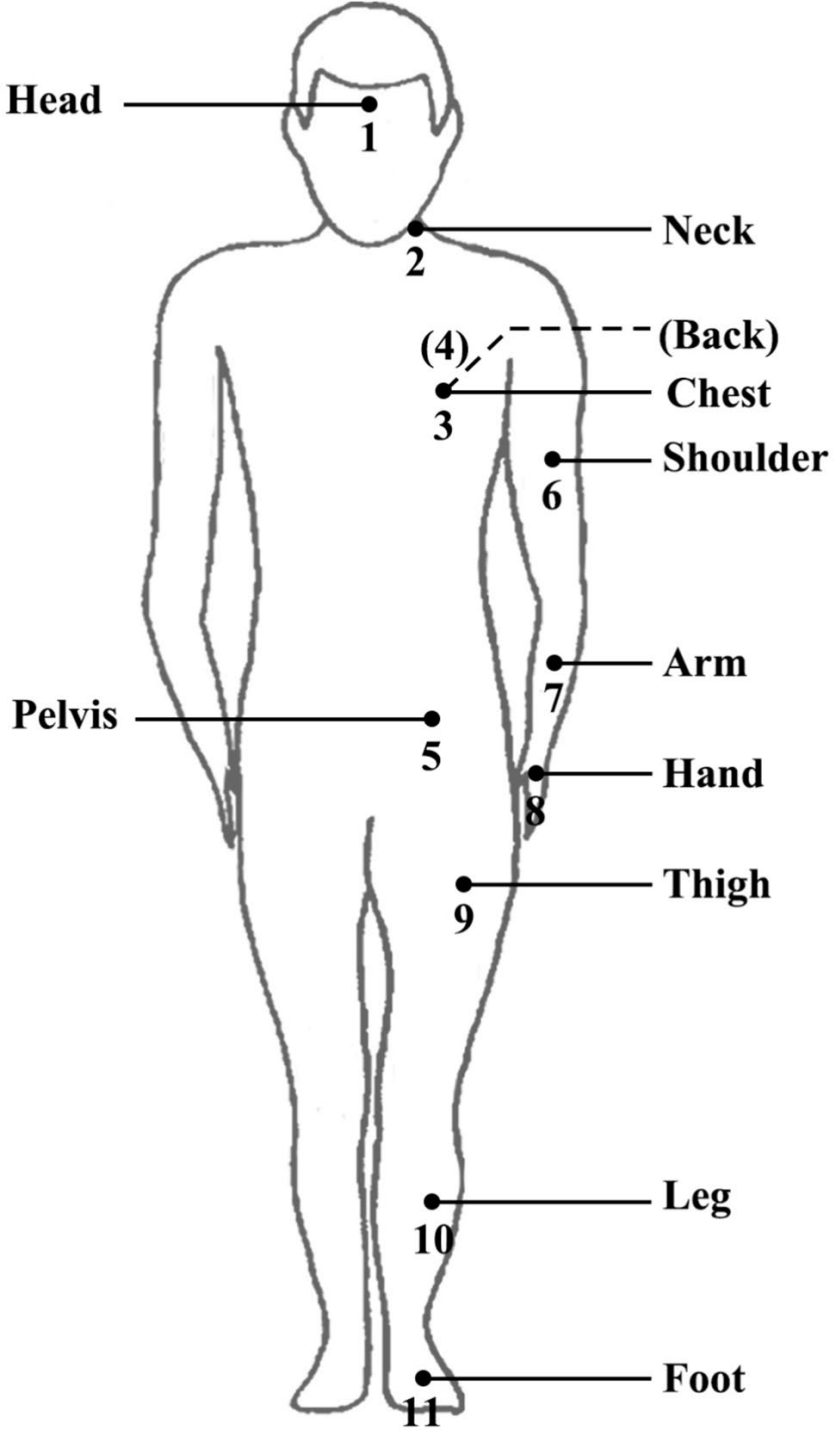
Measurement points of skin temperatures

The experiment procedure is illustrated in Fig. 2. The whole experiment lasted for about 2 h, and subjects experienced alternative exposures to 20-min outdoor environments and 15-min air-conditioned indoor environments for recovery. Throughout the experiment, subjects remained seated on the chairs without backrest in both indoor and outdoor conditions. Using a microclimate station that includes pyranometers (Kipp & Zonen CNR-4, accuracy: < 5%, measurement range: 0–2000 W/m^2^), pyrgeometers (Kipp & Zonen CNR-4, accuracy: < 10%, measurement range: − 250 – + 250 W/m^2^), an anemometer (R.M. YOUNG 81000, accuracy: ± 0.05 m/s, measurement range: 0–40 m/s), and an air temperature and relative humidity sensor (R.M. YOUNG 41382, temperature accuracy: ± 0.3 °C, measurement range: −50– + 50 °C, relative humidity accuracy: ± 1%, measurement range: 0–100%), the environmental parameters were measured. The outdoor environments exhibited a wide range of wind speed levels (around 0.5–2.8 m/s with an average of 1.4 m/s) and mean radiant temperature ranging from 28 to 63 °C, which accounts for overall thermal radiation from various sources. Skin temperatures were measured by thermocouples (KPS-ZT-TT-T-30–1500-CZ, accuracy: ± 1.0 °C, measurement range: −200 – + 200 °C) attaching to subjects’ body segments. A total of 18 thermocouples were used to monitor skin temperatures for different body segments and 11 of them were used for data analysis, as shown in Fig. 3. The measurement points that were not used in the final analysis included the right shoulder, arm, hand, thigh, leg, foot, and face. These points were primarily used for validation, ensuring that the key measurement sites functioned properly and that no significant issues, such as sensor detachment, occurred during data collection. The positions of thermocouples were designed to correspond to the segmentation of thermal manikins and the thermoregulation model, JOS-3 model. Subjects were also asked to complete subjective surveys of thermal sensation vote (TSV) every 2 to 3 min when exposed to the outdoor environment, using the extended nine-point TSV scale (Xie et al. 2019; Zhang et al. 2010). The questionnaires were written in the subjects'native language, Chinese, to facilitate understanding. The reported thermal sensations can help detect extreme thermal conditions, where interactions between highly non-uniform environments and local clothing insulation may occur. By using TSV data, these interactions can be partially decoupled, enabling a clearer assessment of local clothing insulation under outdoor conditions, as detailed in Section "Thermoregulation simulation and model impact assessment".

### Clothing data processing and linear regression

To establish linear regression models and predict local clothing insulation values, the raw clothing data were obtained from various sources with extensive clothing ensemble information, including datasets developed by Lee et al. (2013), Tang et al. (2023) and Smallcombe et al. (2022). These datasets, collected by different institutions across multiple regions, including China, the United States, and Europe, ensure the applicability of the proposed regression models based on them by incorporating diverse clothing patterns. The basic information of these selected datasets is listed in Table 2. Considering that western clothing tends to be mainstream in clothing analysis for thermoregulation and thermal comfort, this study did not include the available non-western dataset (Havenith et al. 2013).

**Table 2:** Description of published clothing datasets

Dataset	Number of ensembles	Position	Sex	Overall clothing insulation range (clo)
Tang et al. (2023)	62	Stand	Male	0.27–2.17
Lee et al. (2013)	40	Sit	Female	0.22–1.24
Smallcombe et al. (2022)	30	Stand	Male	0.31–1.00
	39	Stand	Female	0.25–0.76
	30	Sit	Male	0.28–0.84
	39	Sit	Female	0.23–0.70
Total	240	-	-	0.22–2.17

Since the insulation data for body segments presented in different datasets are not expressed in the same way, these data need to be processed and converted into the same format: local clothing insulation in clo unit (1 clo = 0.155 m^2^·K/W). In Lee’s dataset, they calculated the local clothing insulation values which could be utilized directly (Lee et al. 2013). Differently, Smallcombe et al. (2022) reported regional total insulation values ${I}_{t,i}$, which could readily derive the local clothing insulation values by equations given in ISO 9920 (ISO 2009):

5 $${I}_{cl,i}={I}_{t,i}-{I}_{a,i}/{f}_{cl,i}$$

6 $${f}_{cl,i}=1+0.28\times {I}_{cl,i}$$

where for segment $i$, the local clothing insulation ${I}_{cl,i}$ can be calculated by the regional total clothing insulation ${I}_{t,i}$ and air boundary insulation ${I}_{a,i}$ per clothing area factor ${f}_{cl,i}$ (Eq. (5)). The value of ${I}_{a,i}$ refers to the total clothing insulation measurement on a nude manikin, which could be found in the dataset. The clothing area factor ${f}_{cl,i}$ is related to the local clothing insulation, calculated by the empirical formula, Eq. (6).

Similarly, partial data in Tang’s dataset were recorded as the effective local clothing insulation, ${I}_{clu,i}$, instead of ${I}_{cl,i}$ (Tang et al. 2022, 2020). Fortunately, according to the definition of effective local clothing insulation (ISO 2009), the regional total clothing insulation could be calculated as:

7 $${I}_{t,i}={I}_{clu,i}+{I}_{a,i}$$

The values of ${I}_{cl,i}$ were subsequently derived following the same procedures as what was used for processing data measured by Smallcombe et al. (2022).

Not only the form of the insulation values but also the segmentations vary across datasets due to the use of different thermal manikins. To ensure consistency in segmentations, the body segments involved in the on-site experiment in this study served as the baseline, including the head, chest, back, pelvis, shoulders, arms, hands, thighs, legs, and feet. Since the manikins in selected studies were operated in the uniform surface temperature (Lee et al. 2013; Smallcombe et al. 2022; Tang et al. 2023), the parallel method was used to calculate the regional total insulation values for the target body segments (${I}_{t,{i}{\prime}}$) that include several original segments (Xu et al. 2008):

8 $${I}_{t,{i}{\prime}}={A}_{{i}{\prime}}/\sum {A}_{i}/{I}_{t,i}$$

where ${A}_{{i}{\prime}}$ and ${A}_{i}$ refer to the segmental areas of revised and original body segments, respectively. The revisions of segmentations were applied before the conversion from total insulation values to local insulation values. The detailed relationships between target and original segmentations are provided in Appendix B.

Using the methods outlined above, local clothing insulation data were uniformed for regression analysis. Linear regression models, using a least-squares approach, were employed to determine the line of best fit (Jekel & Venter 2019). In these linear models, overall clothing insulation values were used as the independent variable, while local clothing insulation values served as the dependent variable. The predicted local clothing insulation values were not expected to be lower than those of the thinnest clothing ensemble in the dataset. Thus, referring to the lowest measured local clothing insulation values presented in the dataset, a limit was introduced to each regression function. The general form of linear regression functions is expressed as:

9 $${I}_{cl,i}=\underset{\phantom{0}}{\text{max}}\left({a\bullet I}_{cl}+b, c\right)$$

where ${I}_{cl,i}$ is the local clothing insulation for segment $i$, $a$ is the slope of the independent variable ${I}_{cl}$, $b$ is the vertical intercept, and $c$ is the minimum threshold. When a simple linear model could not adequately capture the relationship between overall and local clothing insulation, attempts were made to fit a piecewise linear regression model instead. The selection of linear or optimized piecewise functions effectively controlled the excessive growth of prediction values for high overall clothing insulation value, unlike the exponential increase often associated with polynomial functions.

### Thermoregulation simulation and model impact assessment

The distribution of local clothing insulation for different body segments is expected to contribute to the prediction accuracy of the existing thermoregulation model. To confirm the validity of the proposed novel clothing model, it is essential to employ a thermoregulation model that accommodates multi-nodal inputs. While many multi-nodal thermoregulation models thoroughly explain their calculation processes, they often lack user-friendly operation. Fortunately, one of the advanced models, the JOS-3 model (Takahashi et al. 2021), could be accessed by its open-source Python codes (https://github.com/TanabeLab/JOS-3). This model was accordingly selected to integrate with proposed local clothing insulation prediction methods. By comparing the simulated skin temperature outputs when using both whole-body and detailed local clothing insulation against the field-collected skin temperature data, the contribution of our clothing prediction model to the accuracy of thermoregulation simulations could be evaluated.

Although most validations of thermoregulation results with clothing insulation estimation methods focus on controlled steady indoor environments (Takahashi et al. 2021), these results might not be sufficient for dynamic environments, especially outdoors. To ensure that the proposed local clothing insulation methods are robust and applicable to real-world scenarios, field experimental data reflecting the responses of the subject in outdoor environments was used, despite the inherent challenges in controlling these conditions. The dynamic nature of outdoor environments, however, complicates efforts to distinguish the effects of local clothing insulation from those of spatial variations in environmental factors such as solar radiation and wind velocity. These factors, similar to clothing insulation, are specific to different body segments and influence them in distinct ways, particularly outdoors. These body-segment-specific influences are difficult to accurately describe and exclude when our field measurements were conducted using a weather station for microclimate condition measurement. In addition, extreme asymmetric local thermal conditions have a significant effect on overall thermal sensations (Zhang et al. 2010), and some of them differ between indoors and outdoors. There are significant differences in thermal sensation between indoor and outdoor conditions when the thermal sensation is away from thermal neutrality according to previous studies (Liu et al. 2022). Therefore, addressing the impacts of these spatially heterogeneous factors is crucial for validations conducted in this study.

To cover the scenarios in outdoor environments while avoiding the complex influence of highly asymmetric conditions, this study examined and compared the ranges of local skin temperatures when subjects experienced thermal neutrality (overall TSV between −0.5 and 0.5), with results from enhanced thermoregulation simulations incorporating the novel local clothing methods. Focusing on periods when subjects voted a neutral sensation can help minimizing the effect of such non-uniform factors, naturally excluding extreme scenarios.

To quantitatively assess the difference between the temperature ranges obtained from simulations and experiments, the Jaccard similarity coefficient (Jaccard 1912) was chosen as an indicator of model accuracy, which is defined as the ratio of the intersection and union for two sample sets. For comparison between different skin temperature ranges in this study, this coefficient was calculated as follows:

10 $$J\left({RT}_{sk,measured},R{T}_{sk,simulated} \right)=\frac{\left|{RT}_{sk,measured}\cap R{T}_{sk,simulated}\right|}{\left|{RT}_{sk,measured}\cup {RT}_{sk,simulated}\right|}$$

${RT}_{sk}$ is defined as the range of skin temperatures that could be measured or simulated. The Jaccard similarity coefficient ranges from 0 to 1. The larger this index is, the more accurate the thermoregulation simulations are.

The local skin temperature ranges from on-site measurements were compared with those obtained through simulations using the JOS-3 model and the proposed local clothing insulation distribution methods. In the experimental data processing, skin temperature data were selected from subjects who reported feeling thermally neutral, with overall TSV ranging from −0.5 to 0.5. When subjects voted a neutral sensation, they were more likely to be in thermal equilibrium and their skin temperatures tended to stabilize. This state of thermal equilibrium was replicated in the simulation. The corresponding skin temperature ranges can thus serve as a baseline for comparison between physiological simulations with different clothing inputs. To minimize fluctuations and potential errors in skin temperature measurements, the skin temperature ranges were defined as the interquartile ranges of the selected skin temperatures, representing the 25 th to 75 th percentiles of the data.

**Fig. 4:**
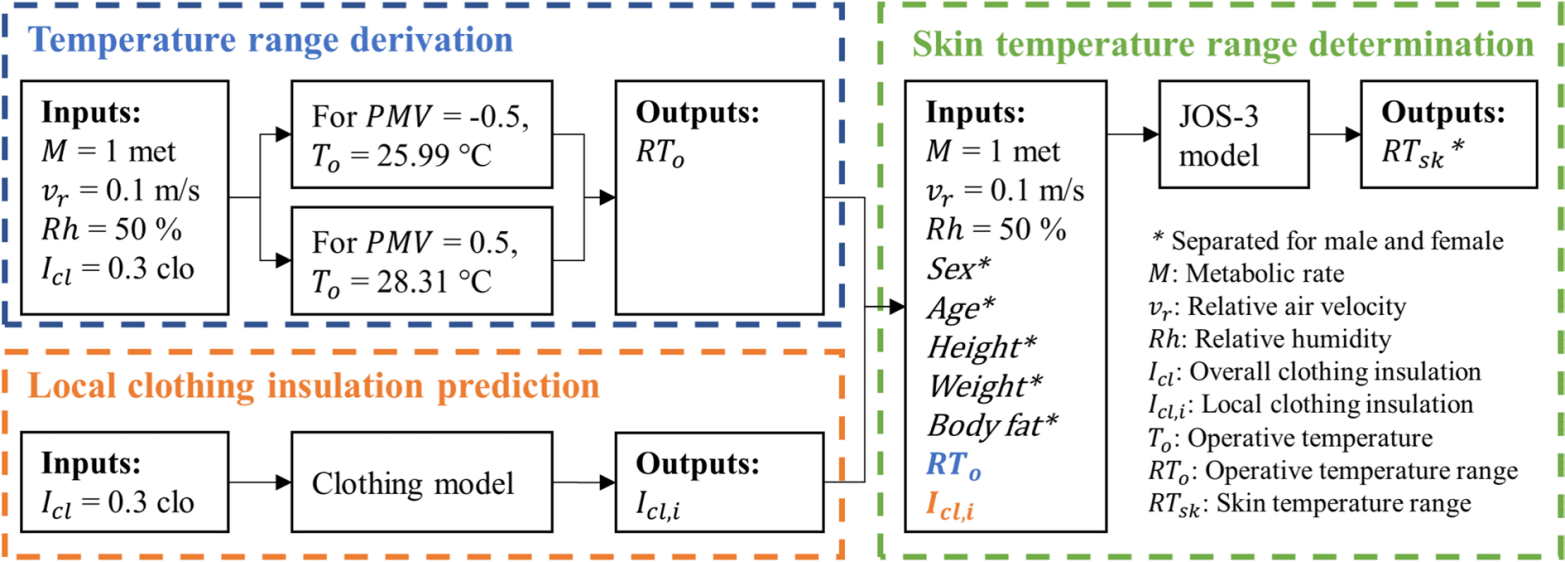
Workflow of thermoregulation simulations

In the simulations, thermal neutrality was defined as the predictive mean vote (PMV) values (Fanger 1972) within ± 0.5. Figure 4 illustrates the workflow for determining simulated skin temperature ranges under thermal neutrality conditions. The process was divided into two main steps: (1) deriving the ambient temperature ranges that could maintain a neutral thermal feeling, and (2) determining the corresponding skin temperature ranges through thermoregulation simulations, using the predicted local clothing insulation values.

The ambient temperature ranges were derived by solving the PMV model. For a given set of input parameters including the operative temperature, air velocity, relative humidity, metabolic rate and clothing level, the PMV could be calculated. By iteratively adjusting the operative temperatures, the PMV model was solved until its output matched −0.5 or 0.5, identifying the corresponding operative temperatures. The remaining physical conditions were set as a typical static indoor environment with air velocity of 0.1 m/s and relative humidity of 50%. Physiological parameters, including a metabolic rate of 1 met and a clothing level of 0.3 clo, were referred to personal information from our field experiments. For these given conditions, the resulting operative temperatures for simulations ranged from 25.99 to 28.31 °C.

These calculated temperatures were then used as inputs for the thermoregulation model, which simulated skin temperature ranges. The period for each simulation lasted 2 h to ensure that results converged to a stable state. Considering the difference in gender, inputs and follow-up simulations were separated for males and females. The thresholds for simulated skin temperatures were defined as the average values of outputs from these gender-specific simulations.

## Results and discussion

### Regression model

**Fig. 5:**
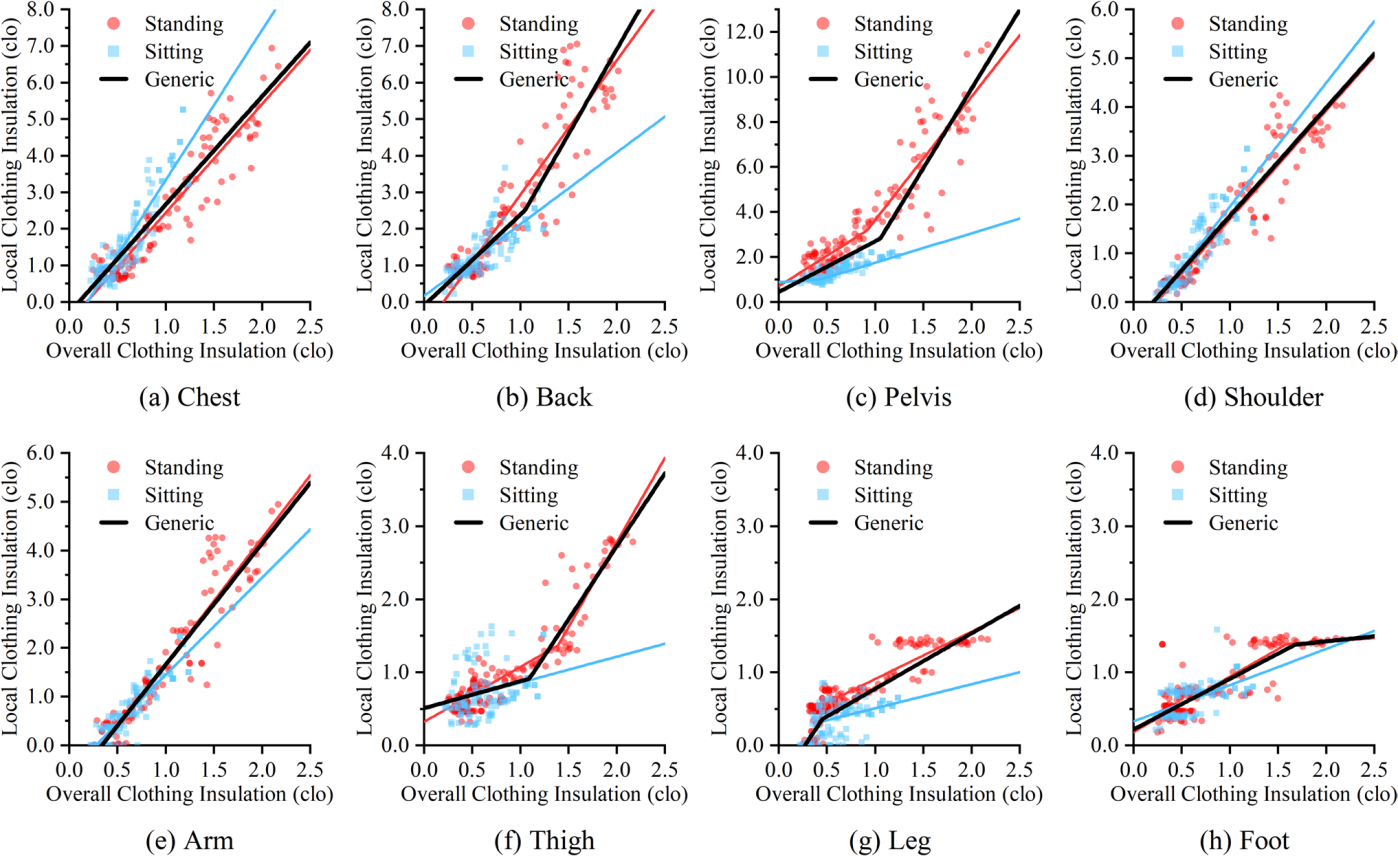
Regression results between overall and local clothing insulation

With the changes in overall clothing insulation, the trend of local clothing insulation values can be demonstrated as linear or piecewise functions. The regression results are shown in Fig. 5 except for values of the head and hands which were being fixed. Due to differences in posture, the regressions were classified into standing, sitting, and a generic category (combining data from standing and sitting postures). Although slight gender differences were observed in underwear and certain dress codes, the models were applied universally for both males and females since the differences were minimal.

These regression models should be interpreted with caution, as the scope of the dataset and surveys was limited. As shown in Table 3, each expression of local clothing insulation has a minimum threshold corresponding to the thinnest clothing ensemble in the selected dataset. Although no maximum limits were defined, the found functions may not be effective for unusually heavy clothing with large insulation (${I}_{cl}$ > 2 clo), such as ski suits and protective suits. These types of clothing can exceed the highest overall insulation values used to develop the regression models, making their local clothing insulation predictions less reliable.

**Table 3:** Regression models of local clothing insulation for different segments

Segment	Equation	Minimum	Position	R^2^
Head	${I}_{cl,i}=0.13$	-	-	-
Chest	${I}_{cl,i}=2.975\times {I}_{cl}-0.540$	0.400	Standing	0.879
	${I}_{cl,i}=4.104\times {I}_{cl}-0.773$	0.415	Sitting	0.814
	${I}_{cl,i}=2.958\times {I}_{cl}-0.297$	0.400	Generic	0.819
Back	${I}_{cl,i}=3.668\times {I}_{cl}-0.738$	0.447	Standing	0.853
	${I}_{cl,i}=1.963\times {I}_{cl}+0.161$	0.220	Sitting	0.605
	$${I}_{cl,i}=\left\{\begin{array}{c}2.483\times {I}_{cl}-0.097, { I}_{cl}<1.050\\ 4.626\times {I}_{cl}-2.347, { I}_{cl}\ge 1.050\end{array}\right.$$	0.220	Generic	0.850
Pelvis	$${I}_{cl,i}=\left\{\begin{array}{c}2.706\times {I}_{cl}+0.698, { I}_{cl}<0.916\\ 5.482\times {I}_{cl}-1.846, { I}_{cl}\ge 0.916\end{array}\right.$$	1.153	Standing	0.853
	${I}_{cl,i}=1.412\times {I}_{cl}+0.514$	0.755	Sitting	0.748
	$${I}_{cl,i}=\left\{\begin{array}{c}2.256\times {I}_{cl}+0.437, { I}_{cl}<1.055\\ 7.047\times {I}_{cl}-4.617, { I}_{cl}\ge 1.055\end{array}\right.$$	0.755	Generic	0.836
Shoulder	${I}_{cl,i}=2.230\times {I}_{cl}-0.534$	0	Standing	0.889
	${I}_{cl,i}=2.510\times {I}_{cl}-0.587$	0	Sitting	0.796
	${I}_{cl,i}=2.221\times {I}_{cl}-0.459$	0	Generic	0.870
Arm	${I}_{cl,i}=2.580\times {I}_{cl}-0.907$	0	Standing	0.892
	${I}_{cl,i}=1.993\times {I}_{cl}-0.548$	0	Sitting	0.810
	${I}_{cl,i}=2.492\times {I}_{cl}-0.840$	0	Generic	0.891
Hand	${I}_{cl,i}=0$	-	-	-
Thigh	$${I}_{cl,i}=\left\{\begin{array}{c}0.752\times {I}_{cl}+0.321, { I}_{cl}<1.390\\ 2.314\times {I}_{cl}-1.849, { I}_{cl}\ge 1.390\end{array}\right.$$	0.316	Standing	0.877
	${I}_{cl,i}=0.354\times {I}_{cl}+0.504$	0.280	Sitting	0.079
	$${I}_{cl,i}=\left\{\begin{array}{c}0.370\times {I}_{cl}+0.507, { I}_{cl}<1.093\\ 1.999\times {I}_{cl}-1.274, { I}_{cl}\ge 1.093\end{array}\right.$$	0.280	Generic	0.777
Leg	$${I}_{cl,i}=\left\{\begin{array}{c}2.794\times {I}_{cl}-0.825, { I}_{cl}<0.505\\ 0.648\times {I}_{cl}+0.260, { I}_{cl}\ge 0.505\end{array}\right.$$	0	Standing	0.856
	$${I}_{cl,i}=\left\{\begin{array}{c}2.057\times {I}_{cl}-0.548, { I}_{cl}<0.420\\ 0.329\times {I}_{cl}+0.178, { I}_{cl}\ge 0.420\end{array}\right.$$	0	Sitting	0.378
	$${I}_{cl,i}=\left\{\begin{array}{c}2.019\times {I}_{cl}-0.552, { I}_{cl}<0.450\\ 0.758\times {I}_{cl}+0.016, { I}_{cl}\ge 0.450\end{array}\right.$$	0	Generic	0.735
Foot	$${I}_{cl,i}=\left\{\begin{array}{c}0.757\times {I}_{cl}+0.182, { I}_{cl}<1.590\\ 0.092\times {I}_{cl}+1.241, { I}_{cl}\ge 1.590\end{array}\right.$$	0.180	Standing	0.777
	${I}_{cl,i}=0.496\times {I}_{cl}+0.327$	0.211	Sitting	0.351
	$${I}_{cl,i}=\left\{\begin{array}{c}0.693\times {I}_{cl}+0.217, { I}_{cl}<1.677\\ 0.139\times {I}_{cl}+1.146, { I}_{cl}\ge 1.677\end{array}\right.$$	0.180	Generic	0.711

According to the results, tops contributed more to overall clothing insulation increase compared to bottoms. The regression functions for body segments affected by tops, including the chest, back, pelvis, shoulders, and arms, are steeper than those for body segments affected by bottoms and footwear, such as thighs, legs and feet. As overall clothing insulation increases, local clothing insulations in core areas (chest, back, and pelvis) increase more rapidly than shoulders and arms among local body segments with steeper slopes. This finding is consistent with a previous study comparing the contribution of upper and lower segments’ clothing insulation values to the total insulation (Nakagawa & Nakaya 2021).

The regression results also reveal differences between sitting and standing postures. Data points for sitting only include cases with overall clothing insulation below 1.5 clo, while standing data covers a broader range. This difference primarily stems from the fact that only Tang et al. (2023) investigated ensembles with higher clothing insulation levels by a standing manikin. The trends in local clothing insulation also differ between sitting and standing, particularly for the chest and pelvis when overall clothing insulation is under 1.5 clo. As compared to standing, local clothing insulation when sitting increases more rapidly for the chest, but slower for the pelvis. This is likely because the sitting posture compresses clothing over certain areas, such as the buttocks, which affects the insulation of both the air boundary and the enclosed air layer. For thick clothing, the sitting posture will reduce the clothing insulation (Nishimura et al. 1994), which may explain the relatively low local clothing insulation observed for the pelvis.

The linear relationship between overall and local clothing insulation is weaker for some body segments when sitting. The regression results show poor linear correlation for lower segments, including the thighs, legs, and feet, making them less accurate for predicting local clothing insulation. This may be due to limited data developed for sitting, with a narrow range of clothing ensembles, mainly pants and footwear, leading to more categorical than linear relationships. Variations in the measurement process for sitting thermal manikins may also contribute to these unexpected regression results. Factors such as the thickness of clothing, types and design of chairs, body surface area in contact with the chair, and inclination of occupants’ torso can all influence the measurements (McCullough 1994). These variations have led to inconsistencies across different studies, making the relationships for sitting posture less clear.

To address weak linear correlation for sitting postures, generic regression models were developed. The fitting lines of these models, colored in black, are presented in Fig. 5. These models used all available data points for analysis, capturing the trends of local clothing insulation changes for sitting postures to some extent. By incorporating both standing and sitting data, these models provided stronger relationships for all body segments. For body regions including the chest and pelvis, where differences between sitting and standing were observed, the generic models offer a balanced approach. Before more comprehensive sitting datasets are available to be included for enhancing the regression analysis, these generalized models serve as a viable alternative.

To verify the regression results, this study compared the predicted local clothing insulation values for clothing ensembles using the proposed methods and those of Tang et al. (2023), based on clothing information collected from the survey. The local clothing insulation values predicted from Tang’s models, which consider detailed garment-level data, tend to be closer to actual conditions. Tang’s models require comprehensive clothing details while our proposed models aim to estimate local clothing insulation using a more streamlined approach that does not rely on detailed ensemble-specific information. If the discrepancies between the predictions of the proposed models and those of Tang’s models remain within an acceptable range, this supports the accuracy and practicality of our approach. A general summary of the prediction results using methods for standing and generic postures is presented in Table 4, including mean values (M) and standard deviations (SD). Compared to Tang’s methods, the proposed methods tended to underestimate local clothing insulation.

**Table 4:** Summary of the local clothing insulation values predicted by different methods

Segment	Predicted by Tang’s methods (standing)		Predicted by linear regression models (generic)		Predicted by linear regression models (standing)	
	M (clo)	SD (clo)	M (clo)	SD (clo)	M (clo)	SD (clo)
Chest	1.319	1.334	1.216	1.006	1.007	0.997
Back	1.375	1.754	1.259	1.123	1.139	1.248
Pelvis	2.634	1.855	1.781	1.397	2.226	1.382
Shoulder	0.943	0.926	0.677	0.756	0.607	0.759
Arm	0.566	1.195	0.461	0.832	0.454	0.854
Thigh	0.818	0.409	0.755	0.328	0.726	0.330
Leg	0.359	0.403	0.319	0.311	0.365	0.360
Foot	1.201	1.040	0.571	0.233	0.568	0.249
Overall	0.516	0.340	0.516	0.340	0.516	0.340

**Fig. 6:**
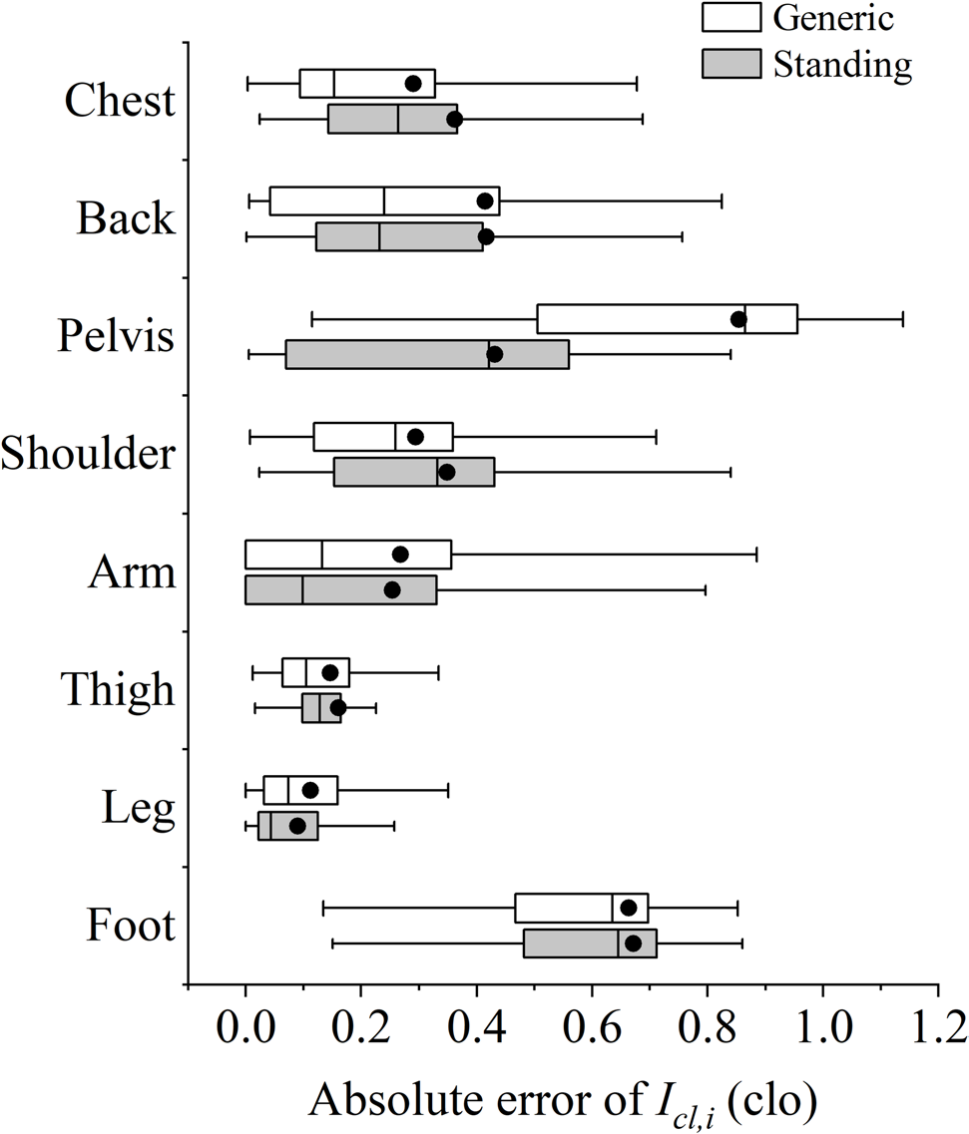
Absolute errors of predicted local clothing insulation values compared to values obtained from Tang’s methods

**Fig. 7:**
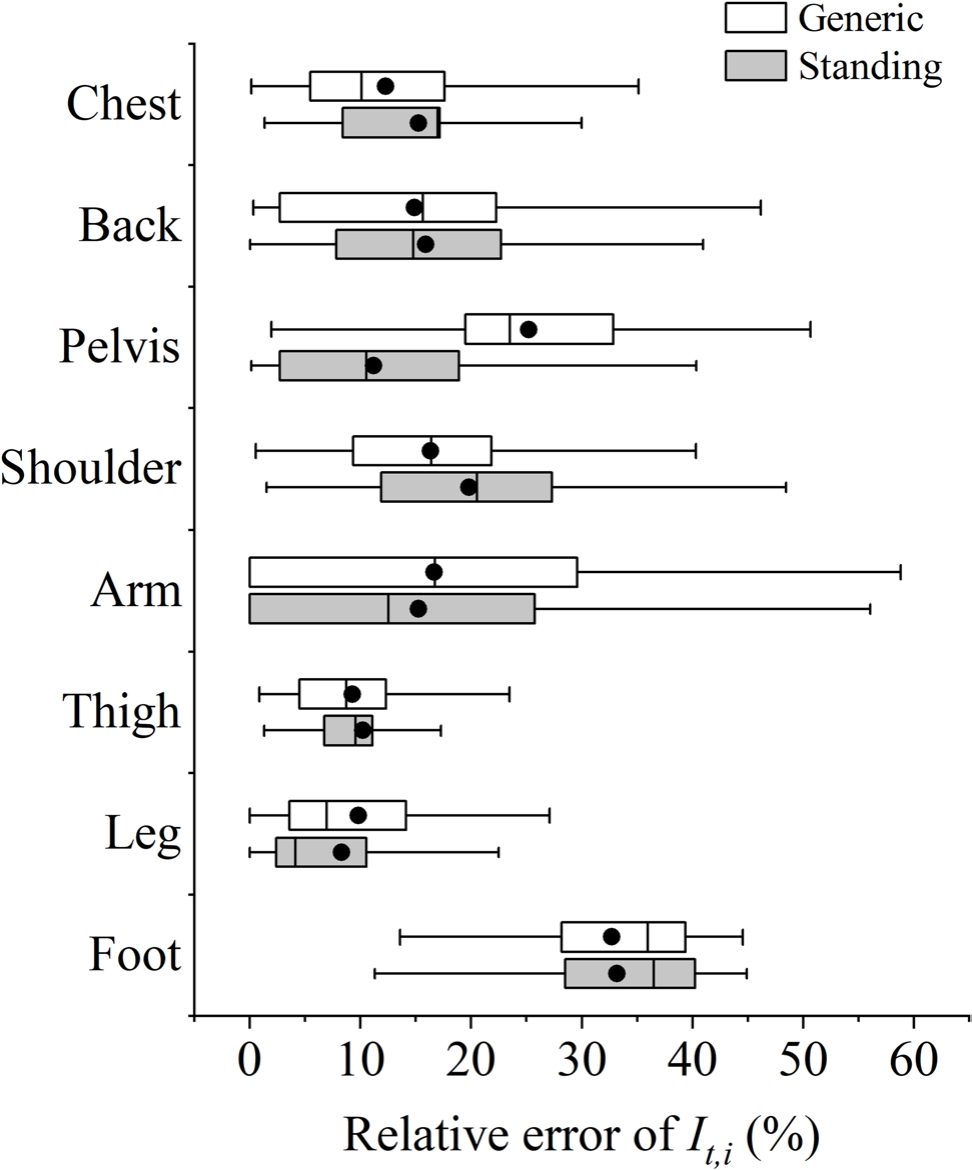
Relative errors of predicted regional total clothing insulation values compared to values obtained from Tang’s methods

Figure 6 shows the absolute error of the proposed methods for standing and generic postures, using Tang’s model predictions as the baseline. The estimation methods developed by Tang et al. (2023), which accounted for the contribution of individual garments, were considered more accurate and thus selected for comparison. The predictions of local clothing insulation for the arms, legs, and thighs showed the best performance among all body segments, with most absolute errors below 0.2 clo. Although the errors were larger for the chest, back, and shoulders, the bias was considered acceptable since the local clothing insulation values were also higher in these areas. The relative errors for these body segments remained below 20%, as shown in Fig. 7. To prevent instances where the denominator in the relative error calculation might equal zero, the comparison used regional total insulation values instead of local clothing insulation values.

Certain body segments exhibited more considerable errors. The predicted local clothing insulation values for the pelvis from the proposed generic methods showed a significant difference compared to those obtained from Tang’s methods, with an average absolute error of above 0.8 clo. Including data related to sitting posture in the generic methods was the main reason for such large difference, since Tang’s methods were designed for standing posture only. Instead, the proposed methods developed for standing posture accordingly performed better and yielded more acceptable results for the pelvis. The large differences were also observed in feet, which may be attributed to subjective preferences in footwear choices. In the clothing survey, subjects often preferred sports shoes with high thermal insulation, even when wearing thin clothing ensembles. The influence of subjective footwear preferences is further discussed in the discussion section.

### Model validity assessment

**Fig. 8:**
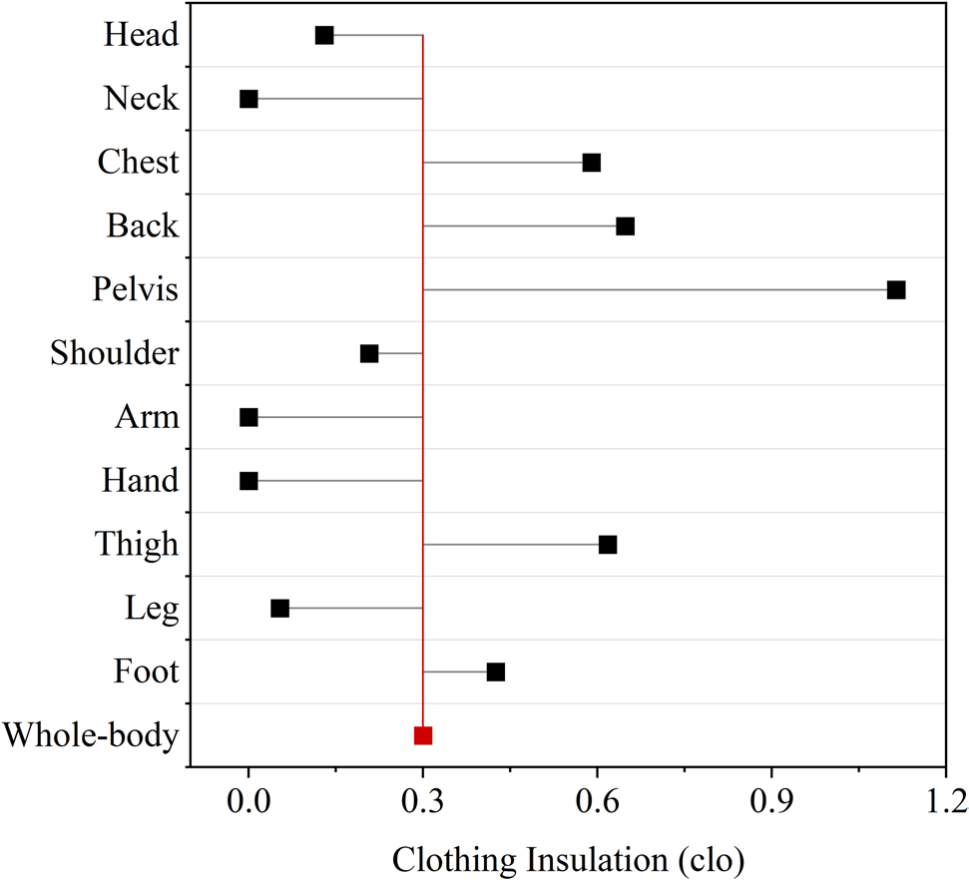
Clothing insulation for different body segments when overall clothing insulation is 0.3 clo

To assess the validity of the proposed methods with regression models, the initial input used the overall clothing insulation of 0.3 clo, which was aligned with clothing choices in the experiment (Table 1). The predicted distribution of local clothing insulation was obtained by proposed regression functions, as shown in Fig. 8. Since subjects remained in sitting posture during the experiment, the prediction methods for generic posture were used. Although the generic models were not completely designed for the sitting posture, they were established based on datasets including clothing data for sitting conditions. Furthermore, for lightweight clothing, the impact of posture on local insulation is relatively minor. The generic models can accordingly represent the actual clothing that subjects wore in the experiment. The clothing insulation value which is not covered by the proposed methods, i.e., the value for the neck, was empirically set to 0 for the JOS-3 simulations.

**Fig. 9:**
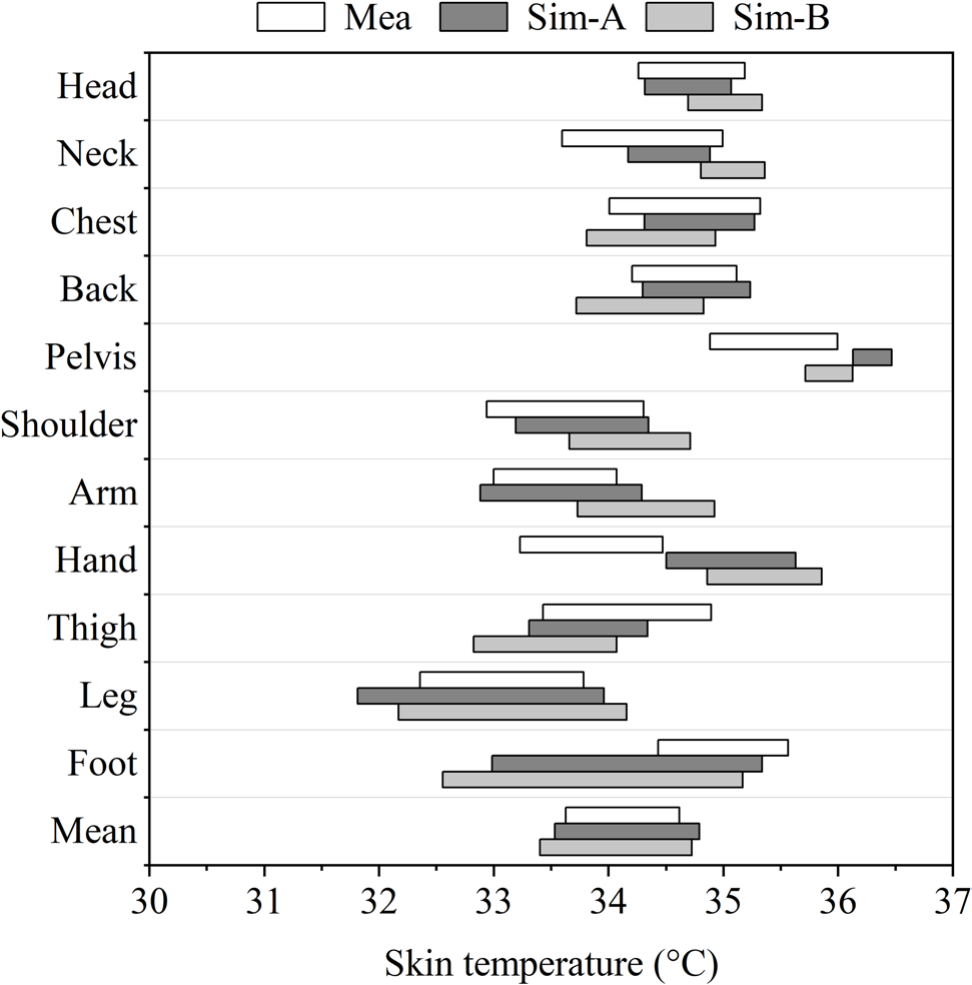
Skin temperature range comparison between measurement data and simulation results using local and overall clothing insulation

Figure 9 shows the neutral skin temperature ranges obtained from measurements and simulations for different body segments. In the figure, the abbreviations “Mea” and “Sim-” refer to measurement and simulation data respectively. The suffixes following “Sim-” are designed to make a distinction between simulations with different settings. The terms “A” and “B” presented here represent results obtained from simulations using multi-nodal local clothing insulation and single overall clothing insulation, respectively. In Fig. 9, the large differences between Sim-A and Sim- B in skin temperature ranges can all be seen in body segments where local clothing insulation is significantly different from overall insulation, including head, neck, chest, back, and arms.

**Fig. 10:**
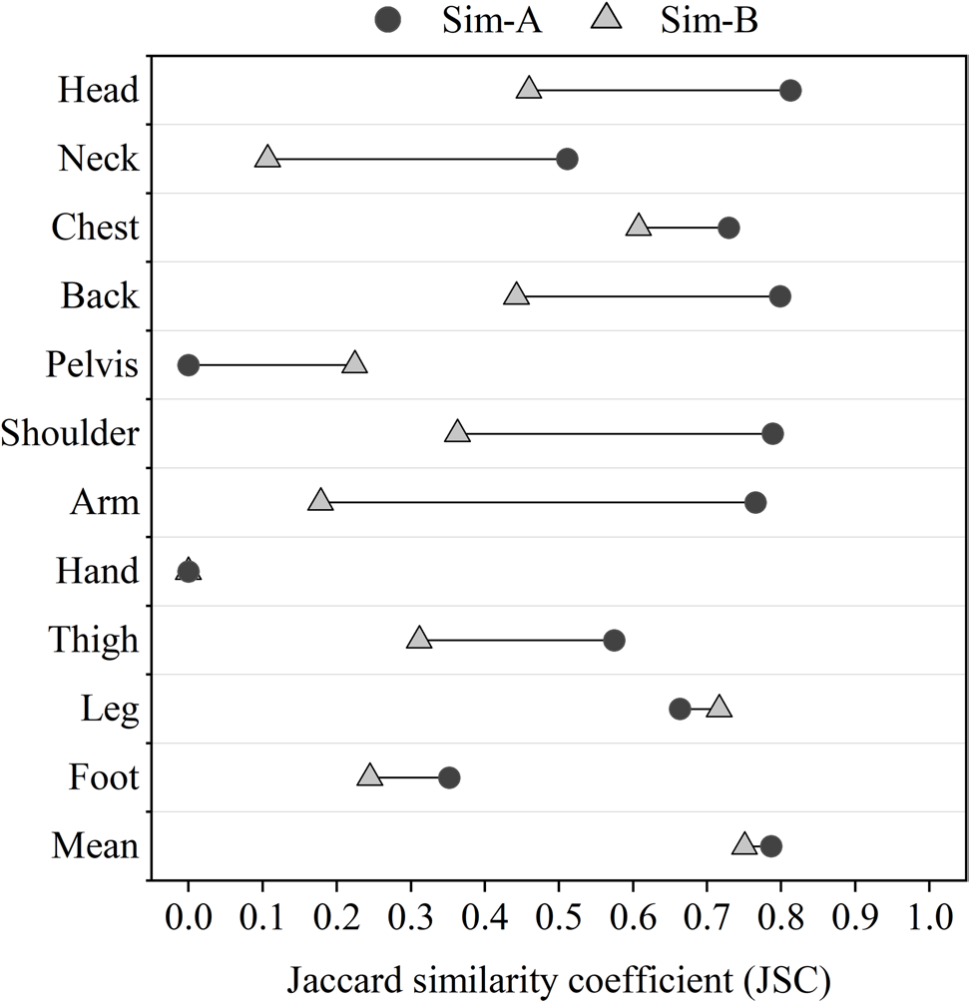
Jaccard similarity coefficient (JSC) comparison between simulation results using local and overall clothing insulation

When compared with measurement data, the JSC values for simulation results that used overall clothing insulation and predicted local clothing insulation as inputs are presented in Fig. 10. From Fig. 10, it can be seen that for most body segments, the usage of detailed local clothing insulation provided more accurate prediction of real skin temperature ranges than simulations with overall clothing insulation. The JSC values for mean skin temperatures and local skin temperatures in head, chest, back, shoulders and arms could achieve above 0.7, which shows satisfied agreement with measured data. For other body segments including the neck, thighs and legs, the corresponding similarity was also well improved and became acceptable by using the proposed clothing model. The results for the pelvis, hands and feet, however, show poor similarity with measurement in both kinds of simulations. The skin temperatures in the feet are obviously lower than the measurement while those in the pelvis and hands are unexpectedly high. The inaccuracy in the hands was mainly due to a range shift, while for the pelvis and feet, issues with overly narrow or wide temperature ranges were also observed. These inconsistencies may be due to various reasons such as the inaccuracy of the chosen thermoregulation model, the influence of outdoor wind environments and subjects’ clothing preferences. The possible explanations for observed inaccuracy will be fully discussed in the next section.

Although discrepancies between measured local skin temperatures and simulation results persist even when using local clothing insulation, simulations incorporating local clothing insulation exhibited much better prediction accuracy compare to those using overall clothing insulation. In stable thermal environments, such as typical indoor settings, the accuracy of the thermal comfort evaluation based on the physiological simulations may not be significantly affected since the simulated mean skin temperature remain similar. However, in highly non-uniform and transient outdoor environments, inaccurate local skin temperature predictions may amplify errors introduced by environmental non-uniformity. These findings indicate that the used of local clothing insulation obtained from the proposed clothing models can enhance the accuracy of thermal comfort evaluations, particularly in outdoor conditions where localized interventions are critical.

### Limitations of the selected thermoregulation model

Among all calculated skin temperature ranges, the pelvis and hands show the lowest similarity with measured data. The inconsistency that occurred in the pelvis and hands can be attributed to different reasons.

**Fig. 11:**
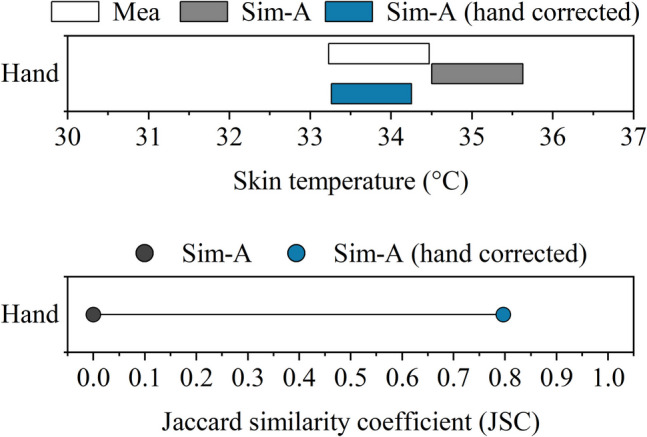
Skin temperature range and Jaccard similarity coefficient (JSC) comparison for hands before and after correction

For the hands, the JOS-3 model introduced the calculation of arteriovenous anastomosis (AVA) blood flow, improving the accuracy of skin temperature prediction (Takahashi et al. 2021). However, the temperature distribution in the hands is highly heterogeneous. With AVAs, the hot blood could be transported to the fingers and palms to adjust body temperatures, especially when staying in the thermoneutral zone (Walløe, 2016). As a result, these areas often have higher skin temperatures. When considering AVAs, the simulated skin temperatures better represent the temperatures in the parts where the AVAs are abundant, i.e., nail beds and palms. Since the thermocouples were placed on the back of the hand during the experiments, the simulated data might differ from what was measured. The inconsistency observed in the findings supports this possible explanation. To correct the dorsal skin temperatures, the relation between dorsal and palmar skin temperatures for the carpometacarpal area, as identified by Leijon-Sundqvist et al. (2017), was applied in this study using the following equation:

11 $${T}_{D}=0.88{T}_{P}+2.9$$

where ${T}_{D}$ and ${T}_{P}$ are skin temperatures for the dorsal and palmar sides, respectively. After this correction, the skin temperature results for hands are much better aligned with the measured dorsal temperatures, as shown in Fig. 11.

**Fig. 12:**
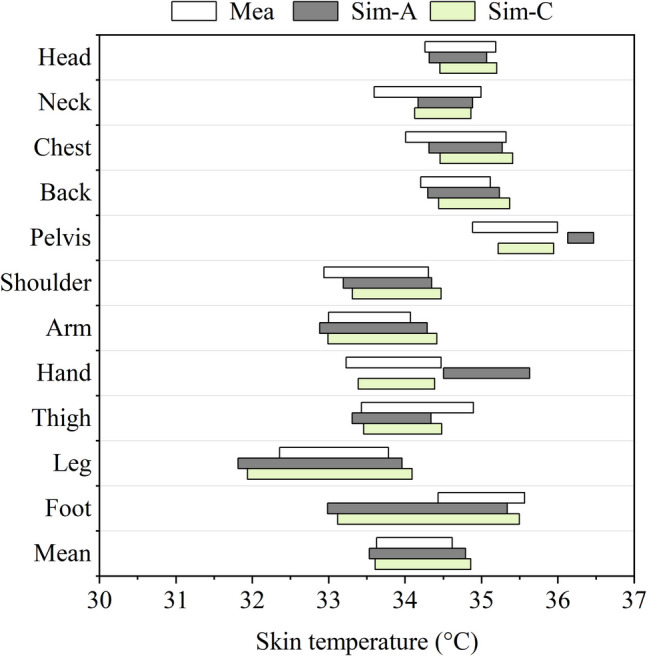
Skin temperature range comparison between measurement data and simulation results using different body construction

**Fig. 13:**
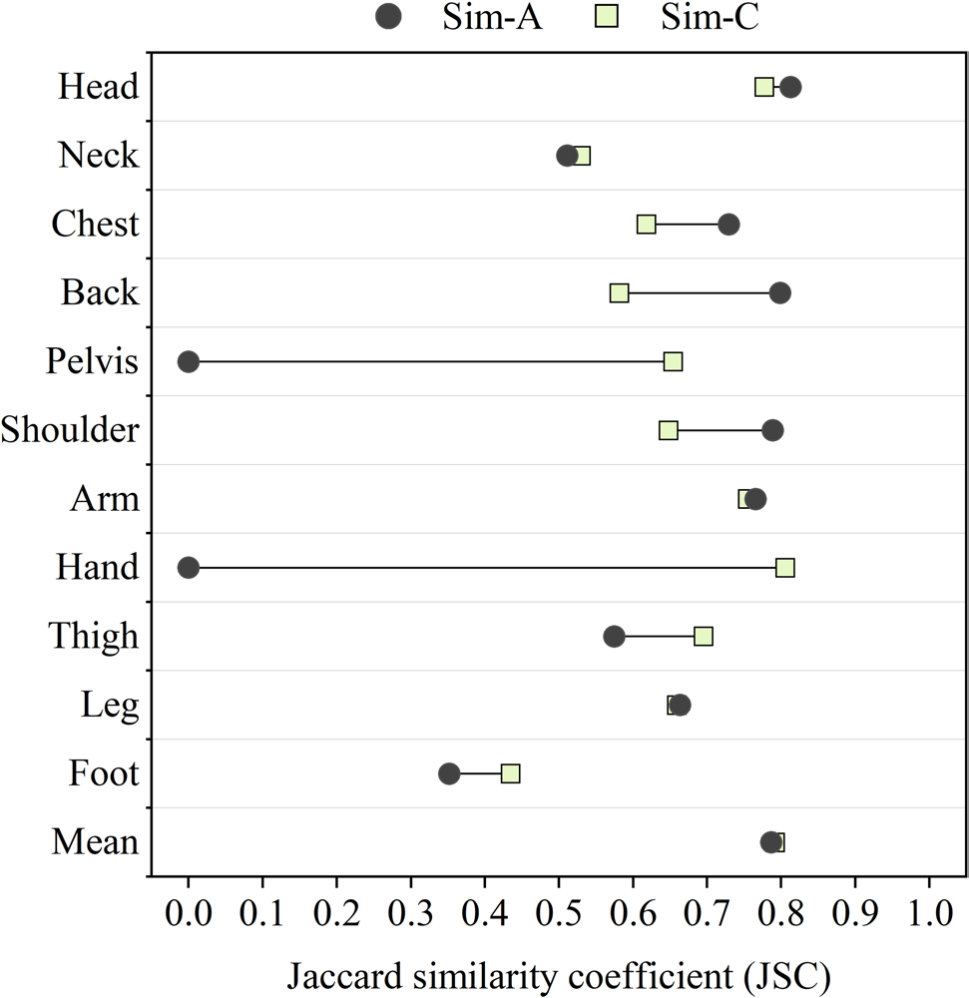
Jaccard similarity coefficient (JSC) comparison between simulation results using different body construction

Similarly, the simulated skin temperatures for the pelvis were significantly higher than the measured values. This discrepancy could be due to the design of the JOS-3 model. Unlike the previous JOS-2 model (Kobayashi and Tanabe 2013), the developed JOS-3 model represents the pelvis with 4 layers (core, muscle, fat, and skin) instead of just two layers (core and skin). Other body regions in the model remained 2-layer. The additional layers for the pelvis may have contributed to the unexpectedly high skin temperatures and reduced temperature fluctuation. To evaluate the effect of body construction on simulation results, the model of the pelvis was modified and rolled back to a 2-layer structure. The results from the original and modified JOS-3 model are compared in Fig. 12 and Fig. 13. Sim-C refers to outputs obtained from the JOS-3 model modified to match the JOS-2 model's structure, including the correction for the hands. What stands out in the figures is the decrease in the pelvis skin temperatures and the wider range of them after the modification. The JSC value for the pelvis accordingly grows from 0 to 0.65, confirming that the 4-layer thermoregulation model contributed negatively to the similarity of pelvis skin temperatures. The skin temperature results in all other body segments slightly increased while that in the pelvis was in the acceptable range.

These results raise concerns about the current JOS-3 model since it did not provide satisfied outputs for different body segments. In the original simulations, temperature results in the hands and pelvis showed much poorer agreement with the measured data than anticipated. While reverting the pelvis to a 2-layer construction improved accuracy, it introduced bias in other regions. To develop the model for more accurate results, establishing 4-layer body constructions for all segments could be a potential solution.

### Correction for clothing insulation under airflow conditions

The wind environment can significantly influence local clothing insulation. The simulation results presented above assumed local clothing insulation values without considering air movement, which may have led to inaccuracies since relatively high wind speeds were observed during the experiment. To better align the predictions with real-world conditions, the effect of wind needs to be evaluated and incorporated into the model.

To adjust local clothing insulation for airflow, many researchers have conducted wind tunnel tests using thermal manikins. One of the datasets selected for this study, Smallcombe’s clothing dataset, provides local clothing insulation values at air velocities of 0.4 m/s and 1.0 m/s (Smallcombe et al. 2022). While these data are readily accessible, they are intended for use with those specific wind speeds or those close to them. For other customized air velocities, Oguro et al. (2002) developed a set of natural logarithmic functions for different body segments to correct the local clothing insulation. Their study helps clarify the impact of relative air velocity on local clothing insulation.

Wind direction also plays a crucial role in real-world wind environments. In the dynamic outdoor wind environment, the wind directions continuously change, making their influence on clothing difficult to assess. Despite these challenges, analyzing wind directions can yield valuable insights. In this study, two directions, upwind and downstream, were examined using correction methods from Oguro et al. (2002).

**Fig. 14:**
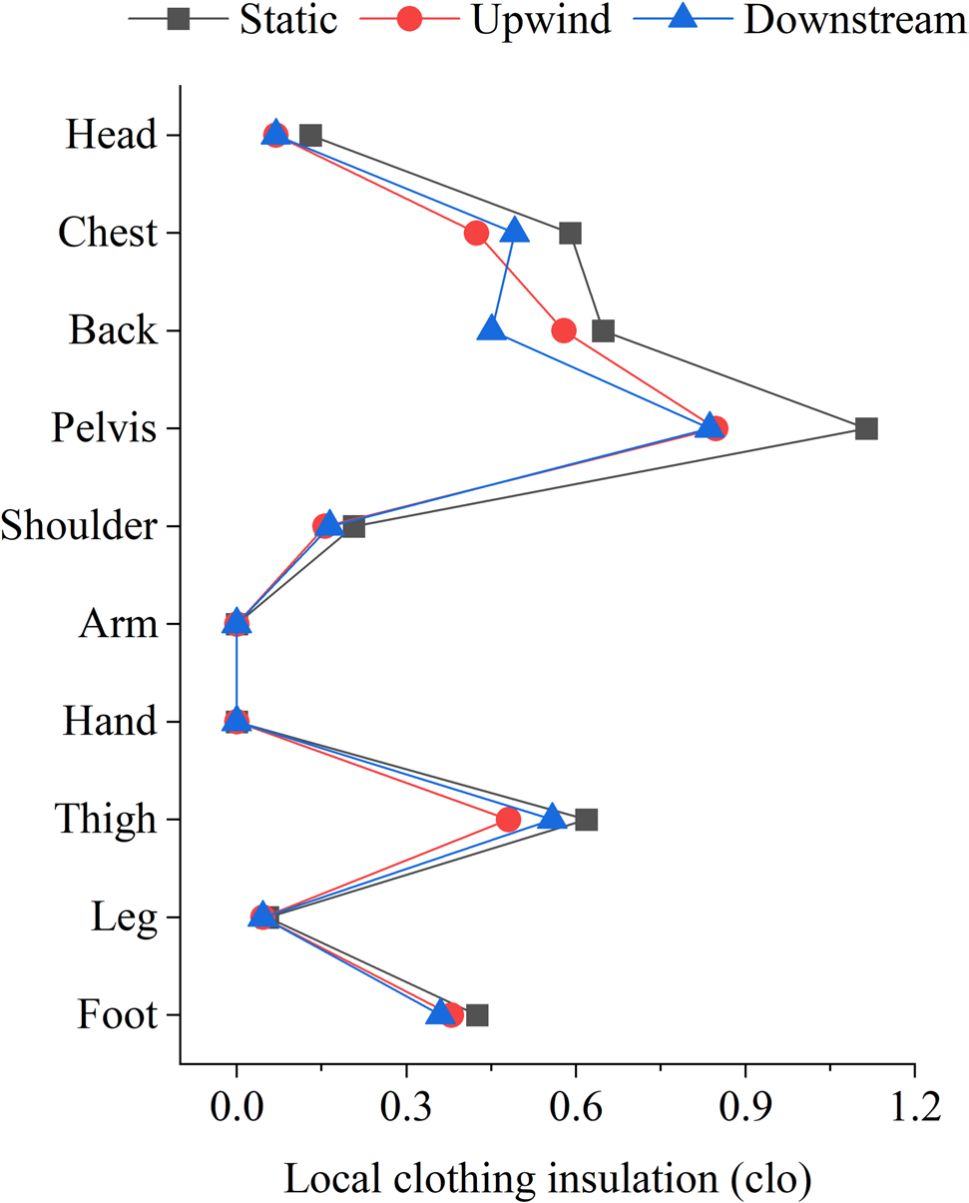
Local clothing insulation values under different wind conditions

Figure 14 shows the corrected local clothing insulation values, using the average measured wind speed of 1.4 m/s, separated by wind direction. It can be observed that local clothing insulation decreased under wind conditions. Downstream wind had a more pronounced effect on the back's insulation compared to upwind, but it was less significant for the chest and thighs.

**Fig. 15:**
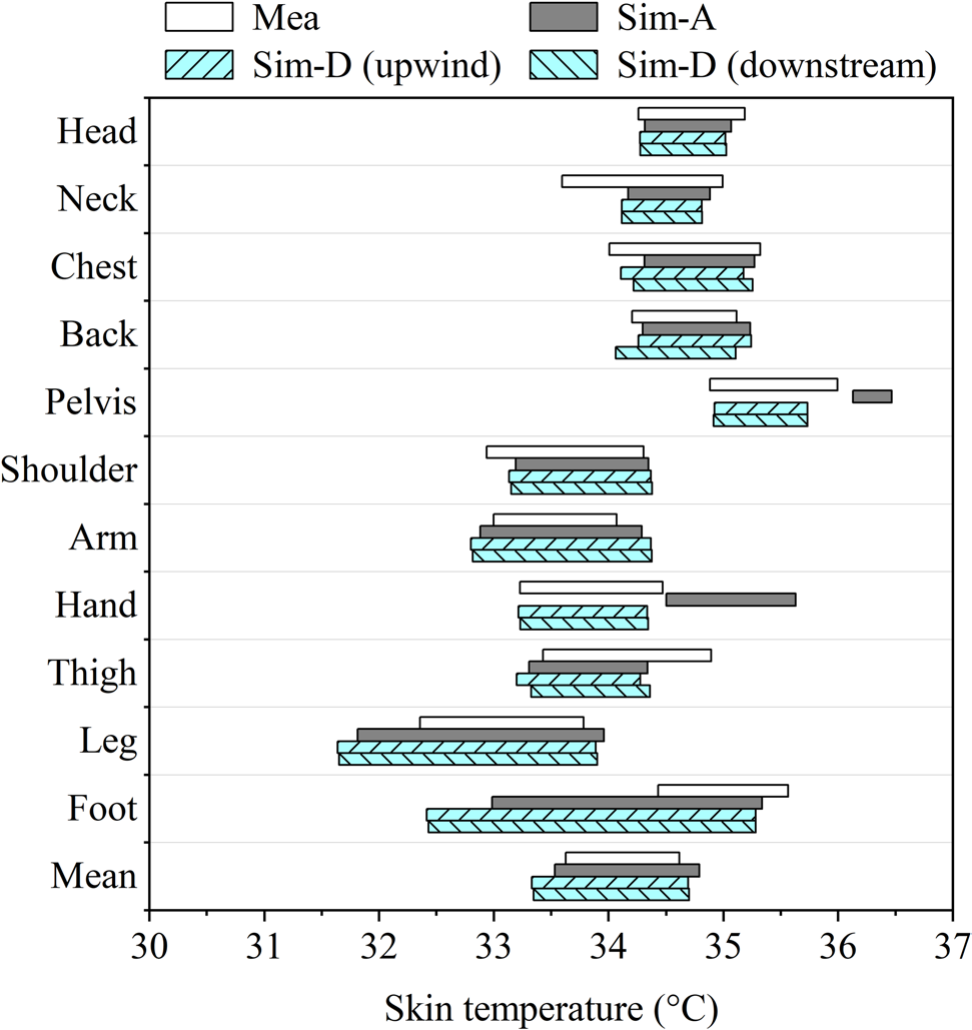
Skin temperature range comparison between measurement data and simulation results considering different wind conditions

**Fig. 16:**
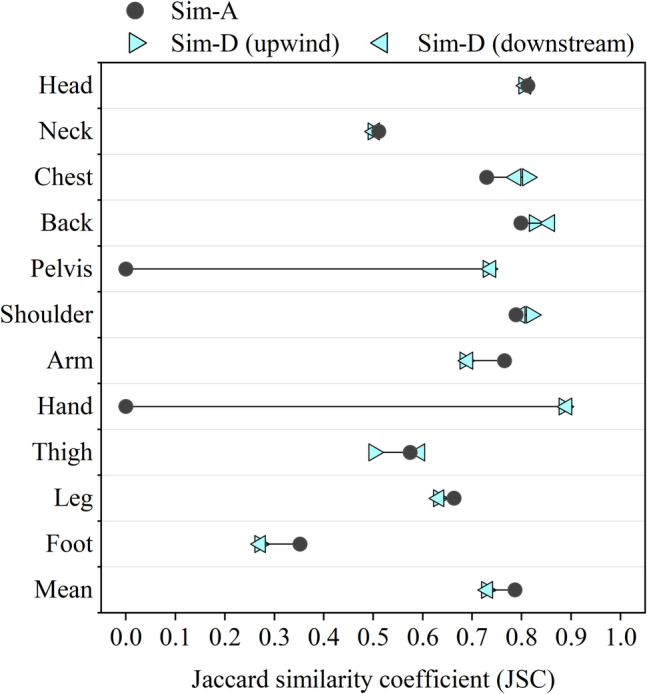
Jaccard similarity coefficient (JSC) comparison between simulation results considering different wind conditions

Figure 15 and Fig. 16 display the thermoregulation simulation results incorporating these corrected insulation values, classified as Sim-D for upwind and downstream conditions. Instead of using the original JOS-3 model, the modified model (2-layer construction) was used for Sim-D. The reduction in local clothing insulation due to wind conditions resulted in slightly lower skin temperatures. The corresponding JSC values for the thighs varied between upwind and downstream conditions, while other body segments showed no significant differences. With local clothing insulation when facing downstream, the JSC value for the thighs was higher. Although no significant differences were observed between the results for Sim-A (static environment) and Sim-D (wind conditions), accounting for wind mitigated the negative effects of skin temperature increases caused by modifications in the JOS-3 model, as seen in Fig. 12 and Fig. 13.

While correction methods for local clothing insulation in highly dynamic wind environments remain limited, this section offers a quantitative analysis that incorporates wind effects. The finding indicated that adjusting local clothing insulation for wind conditions can contribute to the accuracy of thermoregulation simulations.

### Influence of clothing preference on clothing insulation

Beyond the complexities introduced by outdoor environmental factors, such as wind effect, individual clothing preferences also significantly affect the accuracy of thermoregulation simulations. After adjustments to the thermoregulation model and the inclusion of wind effects, discrepancies in local skin temperature ranges and their JSC values were minimized, except for those observed in the feet. The poor agreement between predicted and measured skin temperatures for the feet is attributed to an underestimation of local clothing insulation for this area. This underestimation likely stems from the footwear preferences of the subjects in the experiment. For overall clothing insulation of 0.3 clo used in simulations, the predicted local clothing insulation for the feet was 0.425 clo, representing sandals without socks. However, the recorded clothing data from the experiment indicated that most subjects in Hong Kong wore sports shoes, which have a higher insulation value. This trend was also evident in a clothing survey conducted in Hong Kong and Beijing (Fig. 6).

**Fig. 17:**
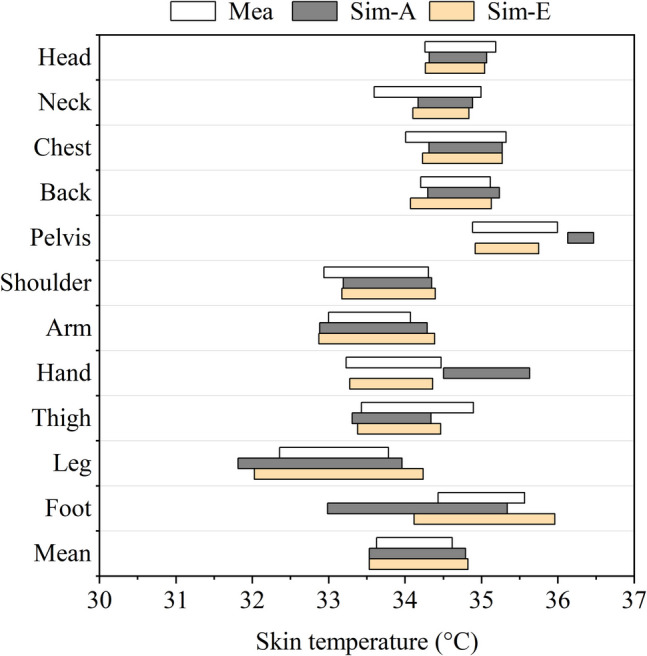
Skin temperature range comparison between measurement data and simulation results considering footwear preference

**Fig. 18:**
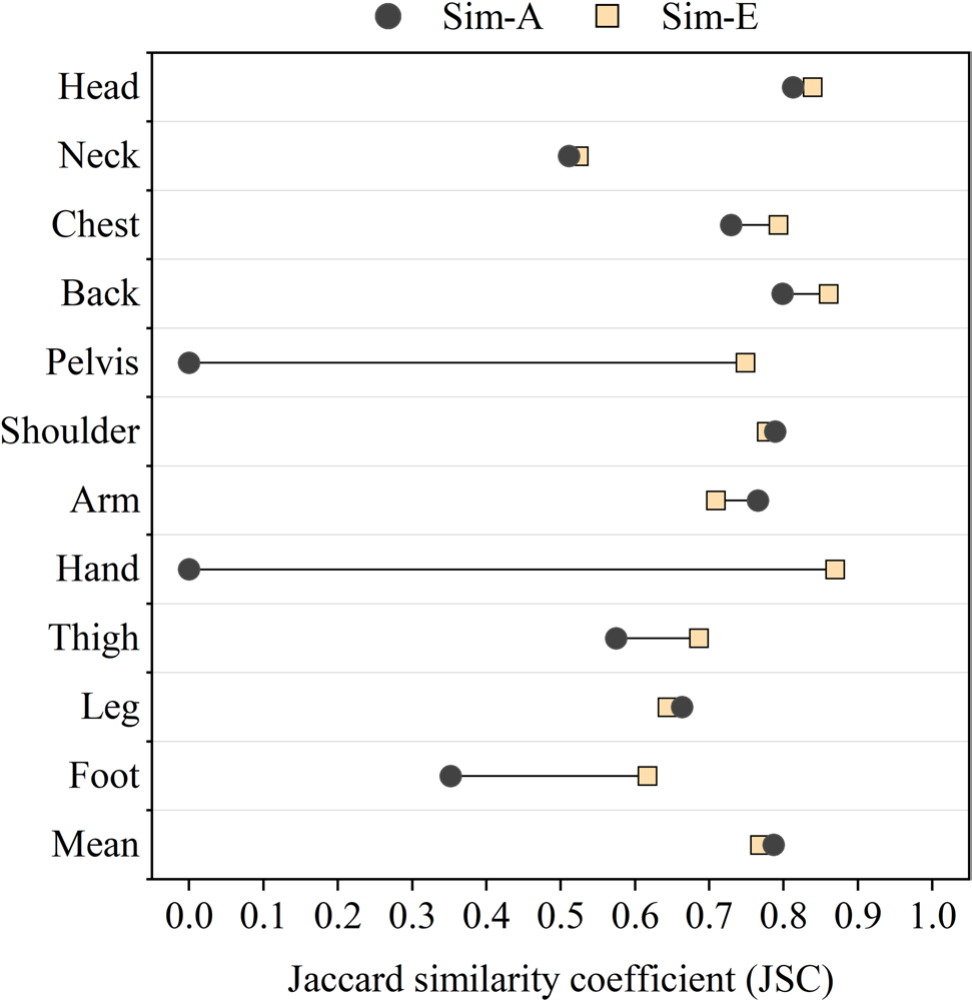
Jaccard similarity coefficient (JSC) comparison between simulation results considering footwear preference

To address this issue, a new local clothing insulation value of 1.580 clo (adjusted to 1.338 clo to account for wind effects), representing sports shoes with ankle socks, was used for the feet. After applying this new value, the results (Sim-E) are presented in Fig. 17 and Fig. 18. All other corrections mentioned were incorporated into the simulation, including adjustments for JOS-3 model configurations and wind effects using methods developed for downstream wind. With these corrections, the predicted skin temperature for the feet increased, leading to a significant improvement in accuracy. The higher foot temperature may have enhanced blood circulation in the lower body segments, thereby raising skin temperatures in the legs and thighs and slightly improving their prediction accuracy.

This adjustment of footwear insulation values highlights a limitation of the proposed methods: they struggle to capture the variety of individual clothing preferences. People's clothing choices may differ from trends observed in published datasets due to factors like culture (Zafarmandi et al. 2024), climate (Salata et al. 2018a, b; Thapa et al. 2024; Yao et al. 2022), psychological effects(Salata et al. 2018a, b), and physiological features (Tiggemann & Andrew 2012). Since individual preferences can significantly influence local clothing insulation predictions, caution should be exercised when using the methods proposed in this study.

## Conclusion

The present study was designed to determine typical local clothing insulation distribution when accurate measurements could not be accessed. Clothing surveys were conducted in Beijing and Hong Kong, China, to gather data on clothing choices. Field experiments were also carried out in Hong Kong to collect physiological parameters and subjective votes for thermal environments. The experimental data integrated with previously published datasets of local clothing insulation provided a deeper insight into how to determine multi-nodal clothing inputs for thermoregulation and thermal comfort models.

The following conclusions can be drawn:

1. Local clothing insulation prediction methods were proposed based on clothing datasets from previous studies. These methods utilize a set of linear regression models for different body segments and require only overall clothing insulation as input. Despite the simplification, the methods demonstrated acceptable agreement with existing estimation methods which need more complicated procedures. The proposed approach allows for the straightforward estimation of local clothing insulation from overall values, leading to more accurate, efficient, and practical simulations of thermal responses and perception.
2. Field measurements of local and mean skin temperatures were used to assess the effectiveness of proposed methods which integrated a detailed local clothing prediction model with a multi-nodal thermoregulation model. Compared to using a single value to represent uneven clothing insulation distribution, the results indicate that the detailed local clothing insulation inputs can increase the similarity between simulated and measured data (the JSC values) by an average of 0.21. For neck, shoulders and arms, particularly, the similarity index, JSC, could be raised by approximately 0.4 and above.
3. Several disturbances to simulation outputs were detected and discussed in terms of thermoregulation model configurations and seated postures. Although the theoretical implications of these findings are unclear, the discussion shows great potential in improving model accuracy. Based on the reasonable assumption, some modifications were made for the used thermoregulation model, JOS-3, and its clothing inputs. The corrected simulation results showed a significant average increase of 0.19 in the JSC values compared to the original outputs. These discussions contribute to extending physiological simulations to more complex conditions in future outdoor thermal comfort studies.

This work contributes to the development of the thermal physiological and comfort model by offering fast and simplified methods for determining typical local clothing insulation and quantitatively evaluating the importance of local clothing insulation in thermoregulation simulations. By supporting the accurate detection of human thermal sensation and comfort, a more precise and easily operated clothing model could be of assistance to architectural or urban design processes, benefiting all city dwellers.

## Figures and Tables

**Fig. 19:**
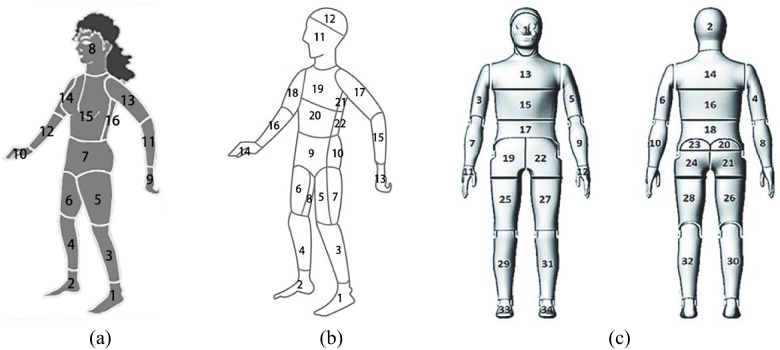
The zone structure and corresponding numbers of manikins used by (**a**) Lee et al. (2013), (**b**) Tang et al. (2020) and Smallcombe et al. (2022)

## Data Availability

Data will be made available on request.

## Appendix B. Depiction of regional zones for manikins

See Fig. 19 and Table 5.

**Table 5:** Correspondence between proposed segmentation and original manikin zones

Segment	Zone Number		
	Lee	Tang	A
Head	8	11, 12	1, 2
Chest	15	19, 20	13, 15
Back	16	21, 22	14, 16
Pelvis	7	9, 10	17, 18, 19, 20*, 21, 22, 23*, 24
Shoulder	13, 14	17, 18	3, 4, 5, 6
Arm	11, 12	15, 16	7, 8, 9, 10
Hand	10, 11	13, 14	11, 12
Thigh	5, 6	5, 6, 7, 8	25, 26, 27, 28
Leg	3, 4	3, 4	29, 30, 31, 32
Foot	1, 2	1, 2	33, 34

* The internal segments
